# Impact of co-fermentation of *Saccharomyces cerevisiae* and *Pichia kluyveri* on the metabolic characteristics of the flavor compounds in mulberry wine

**DOI:** 10.3389/fnut.2025.1559599

**Published:** 2025-02-25

**Authors:** Bo Ding, Ling Xiong, Shutian Zhao, Ying Lin, Penghui Guo, Wenxue Zhang

**Affiliations:** ^1^College of Biomass Science and Engineering, Sichuan University, Chengdu, China; ^2^College of Life Science and Engineering, Northwest Minzu University, Lanzhou, China; ^3^School of Liquor-Brewing Engineering, Sichuan University of Jinjiang College, Meishan, China

**Keywords:** mulberry wine, *Saccharomyces cerevisiae*, *Pichia kluyveri*, metabolic characteristics, co-fermentation

## Abstract

This study investigated the metabolic characteristics of mulberry wine produced by co-fermentation with *Saccharomyces cerevisiae* (SC) and two different *Pichia kluyveri* (PK). Although *S. cerevisiae* inhibited the growth of *P. kluyveri* during co-fermentation, *P. kluyveri* showed robust growth adaptability. Classical oenological parameters were not significantly altered by co-fermentation compared to pure-fermentation. The *P. kluyveri* significantly modulated amino acid metabolism pathways during co-fermentation, enhancing the biosynthesis of higher alcohol acetate compounds. Furthermore, co-fermentation strategy promoted the production of volatile flavor compounds, particularly esters and alcohols, which enriched the wine with distinct floral and fruity flavors. This study provides novel insights into the metabolic mechanisms of co-fermentation with SC and PK strains and highlights the potential of *P. kluyveri* as a co-fermentation agent for improving the aromatic complexity of fruit wines.

## 1 Introduction

Although grapes still dominate the fruit wine market, changing consumer habits mean that other types of fruit wine are gradually coming to the fore (1). Actually, due to their unique flavor and reasonable sugar level, many temperate and tropical fruits have great potential in the new fruit wine industry (2). Mulberry (*Morus alba* L.) is widespread throughout the world, from tropical to temperate areas (3, 4). After alcoholic fermentation, mulberry wine has a complex aroma, elegant taste and a distinctive purple-black color (5). Mulberries consist of clusters of small, juicy drupelets, making them highly susceptible to mechanical damage and resulting in a limited postharvest shelf-life. Consequently, the production of fruit wines represents a viable strategy for enhancing the economic value of mulberries.

In the fermentation process of fruit wine, yeast plays an important role due to its high capacity to produce alcohol and aromatic compounds (6). The yeast mentioned here is, of course, mainly *Saccharomyces cerevisiae*. *S. cerevisiae* is mainly used for its high alcohol production capacity and good tolerance in the traditional fruit wine industry. However, the widespread use of commercial yeasts can lead to homogenization and a lack of flavor diversity in fruit wines made from different raw materials (7). On the other hand, it has been confirmed that some non-*Saccharomyces* are more efficient at producing flavor substances such as esters and other aroma compounds, but less efficient at producing alcohol (7). This is an important factor that limits the industrial use of non-*Saccharomyces* yeasts. As a promising way to increase the flavor diversity of fruit wines, co-fermentation by *S. cerevisiae* and non-*Saccharomyces* has been proposed (8, 9). With this in mind, there is great interest in exploring new co-fermentation strategies using *S. cerevisiae* and different non-*Saccharomyces* yeasts, as these yeasts add complexity to flavors and increase the yield of desirable compounds (10, 11).

Previous report has confirmed that co-fermentation by *Pichia* and *S. cerevisiae* could increases the flavor composition (12). In our previous research, we found the “increased flavor composition” due to the excellent performance of *Pichia kluyveri* in production of esters and higher alcohols (13). This characteristic of *P. kluyveri* has the potential to improve the flavor of fruit wines fermented with materials that lack varietal aroma. Therefore, to achieve effective control and standardization of co-fermentation processes, it is essential to investigate the interaction mechanisms between *S. cerevisiae* and *P. kluyveri* that contribute to the enhancement of wine flavor characteristics.

In this sense, the main objective of this study was to investigate the fermentation quality, yeast cell viability, and metabolic profiles (volatile and non-volatile) in mulberry juice fermentations using pure or co-fermentation with *P. kluyveri* and *S. cerevisiae*. Finally, elucidation of the major impacted metabolic regulation pathways and key metabolites involved in co-fermentation, for a comprehensive evaluation of the effect of the co-fermentation strategy on mulberry wine characteristics.

## 2 Materials and methods

### 2.1 Materials and reagents

Fresh mulberries of “Yun No. 2” (with 12 ± 2 °Bx) were provided by Sixi Agricultural Development Co., Ltd. (Panzhihua, China). All strains used in this study were screened from the “Yanbian” mulberry orchard (Panzhihua, China). The *Saccharomyces cerevisiae* and two different *Pichia kluyveri* were abbreviated as SC, PK1, and PK2, respectively.

Yeast peptone dextrose (YPD) broth medium and Wallerstein nutrient agar (WL) were purchased from Qingdao Hope Bio-Technology Co., Ltd (Qingdao, China). The 4-Methyl-2-pentanol (internal standard for GC-MS) and n-alkanes C6 to C30 (calculate the retention index, RI) were purchased from Sigma-Aldrich Co. (Saint Louis, USA).

### 2.2 Fermentation conditions

Mulberry wine-making followed our previously described method (13). Mulberry juice was prepared by crushing mulberry fruit. Mulberry juice with pectinase (> 500 U/mg) for enzymatic hydrolysis at 20°C for 24 h. Addition of sucrose to pasteurized mulberry juice for standardization (Brix, 25 °Bx). The effective concentration of sulfur dioxide in the mulberry juice was adjusted to 50 mg/L using potassium metabisulphite. The different strains were pre-cultured (28°C, 170 rpm) separately in YPD medium for 40 h. After incubation, resuspend the yeast cells in a sterile physiological saline solution. Each strain was inoculated separately at 10^6^ CFU/mL.

The fermentation groups by SC (*S. cerevisia*), PK1 (*P. kluyveri*), and PK2 (*P. kluyveri*) strains pure-fermented were set up as F(S), F(P1), and F(P2), respectively. The group co-fermented by SC and PK1 strains as group F(S-P1). Similarly, F(S-P2) represented the co-fermentation group of SC and PK2 strains. The fermentation process for each experimental group was terminated when the PK strain was no longer detectable in the co-fermentation.

### 2.3 Microbial counting

During fermentation, yeast counts were carried out every 3 days using WL agar plate (14). One milliliter of fermenting mulberry juice was diluted to 10^−4^ and 10^−5^. Plated on WL agar, incubated at 28°C for 48 h to facilitate separate yeast population counts. The colonies of *S. cerevisiae* (dark green, smooth colony) and *P. kluyveri* (light green, rough colony) could be distinguished by different color and morphology.

### 2.4 Physicochemical analysis

According to previous method (15), soluble solids content (SSC) and pH were monitored every 3 days. The content of SSC was measured by a digital refractometer (BM-04S, Tianjin Nohawk Optoelectronic Technology Co., Ltd, Tianjin, China). The pH was determined using a pH meter (PHSJ-5T, Shanghai INESA Scientific Instrument Co., Ltd, Shanghai, China). The titratable acidity content (TA), the ethanol content and the residual sugars content of the wine were determined at the end of the fermentation process.

### 2.5 Non-volatile component analysis

The Samples (the fermentation broth containing the strains) were thawed at 4°C and vortexed for 1 min to ensure homogeneous mixing. The sample was transferred to a 2 mL centrifuge tube, 500 μL of methanol solution (−20°C) was added. The tube was vortexed for 1 min. Following centrifugation at 12,000 rpm for 10 min at 4 °C, the entire supernatant was transferred to a new 2 mL centrifuge tube for concentration and drying. Subsequently, 300 μL of a solution of 2-amino-3-(2-chlorophenyl) propionic acid (4 ppm) prepared in 80% methanol/water (v/v) was added to the sample. The supernatant was finally filtered through a 0.22 μm membrane, and the filtrate was collected into sample vials for subsequent LC-MS analysis.

Non-volatile component analyzed by Vanquish UHPLC System (Thermo Fisher Scientific, USA) coupled to an Q Exactive Focus (Thermo Fisher Scientific, USA). The connected chromatographic column of choice was of ACQUITY UPLC HSS T3 (150 × 2.1 mm, 1.8 μm, Waters Corp., USA). The temperature of column was kept at 40°C. Flow rate was set at 0.25 mL/min and the volume of the injection at 2 μL. Preparation of four sets of mobile phases, 0.1% formic acid in water (labeled as A1), 0.1% formic acid in acetonitrile (labeled as B1), 0.005 mol/L ammonium formate (labeled as A2) and acetonitrile (labeled as B2). Mobile phases A1 and B1 were used in the positive ion detection mode. Separation gradient programme followed 2% B1 at 0–1 min, 2–50% B1 at 1–9 min, 50–95% B1 at 9–12 min, 95% B1 at 12–14 min, and 95–2% B1 at 14–15 min, 2% B1 at 15–20 min. For negative ion detection mode, the mobile phases A2 and B2 were used. Separation gradient programme followed 2% B2 at 0–1 min, 2–50% B2 at 1–10 min, 50–95% B2 at 10–12 min, 95% B2 at 12–14 min, 95–2% B2 at 14–15 min and at 2% B2 at 15–20 min. The MS/MS parameters were based on previous research (16). It should be noted that the spray voltage set at 2.50 kV and −2.50 kV in positive and negative ion detection mode respectively.

### 2.6 Volatile composition analysis

The volatile compounds of young mulberry wine were analyzed by Headspace solid-phase microextraction-gas chromatography-mass spectrometry (HS-SPME/GC-MS) with some changes based on previous study (17). Briefly, 4 mL of mulberry wine was mixed with 0.4 g NaCl and 10 μL of internal standard (4-Methyl-2-pentyl alcohol, 81.8 μg/mL in water) and placed in headspace vials, then stored at 4°C before analysis. Each sample should be heated 10 min at 60°C before extraction. Volatiles were then extracted from headspace vials using SPME fibers (50/30 μm DVB/CAR/PDMS, Supelco, Bellefonte, PA). The temperature of extraction process was kept at 60°C for 40 min. All samples were analyzed for the presence of volatiles using Trace 1,310 GC (Thermo Fisher Scientific, USA) coupled to a TSQ 9,000 MS (Thermo Fisher Scientific, USA). Selection of a capillary column of the VF-WAXms (30 m × 0.25 mm × 0.25 μm, Agilent Technologies, USA). The adsorbed compounds in the SPME fiber were desorbed in the Trace 1,310 in the splitless mode at 240°C for 10 min. A flow rate of 1.6 mL/min was set for helium as the carrier gas. The oven temperature was set as 40°C for 2 min. The oven temperature was then increased to 50°C at a rate of 4°C/min, followed by an increase to the end point of 200°C at a rate of 5°C/min and held for 5 min to the end of the programme. The analytes were scanned from 30 to 400 m/z using electron impact (EI) ionization mode at 70 eV. The temperature of MS transfer line was set at 200°C and the ionization source temperature was at 220°C. All compounds detected were identified by comparing their retention indices (RI) with those of alkane (C6–C30) and by mass matching against the National Institute of Standards and Technology (NIST) database.

### 2.7 Data analysis

SPSS 24.0 (SPSS-IBM Inc., USA) was used for analysis of variance (ANOVA). The principal component analysis (PCA) was performed using the online tool MetaboAnalyst (https://www.metaboanalyst.ca/). PCA was normalized using Pareto scaling. The heatmap was performed using the online tool Chiplot (www.chiplot.online).

## 3 Results and discussion

### 3.1 Growth of yeasts cells during fermentation

**Figure 1:**
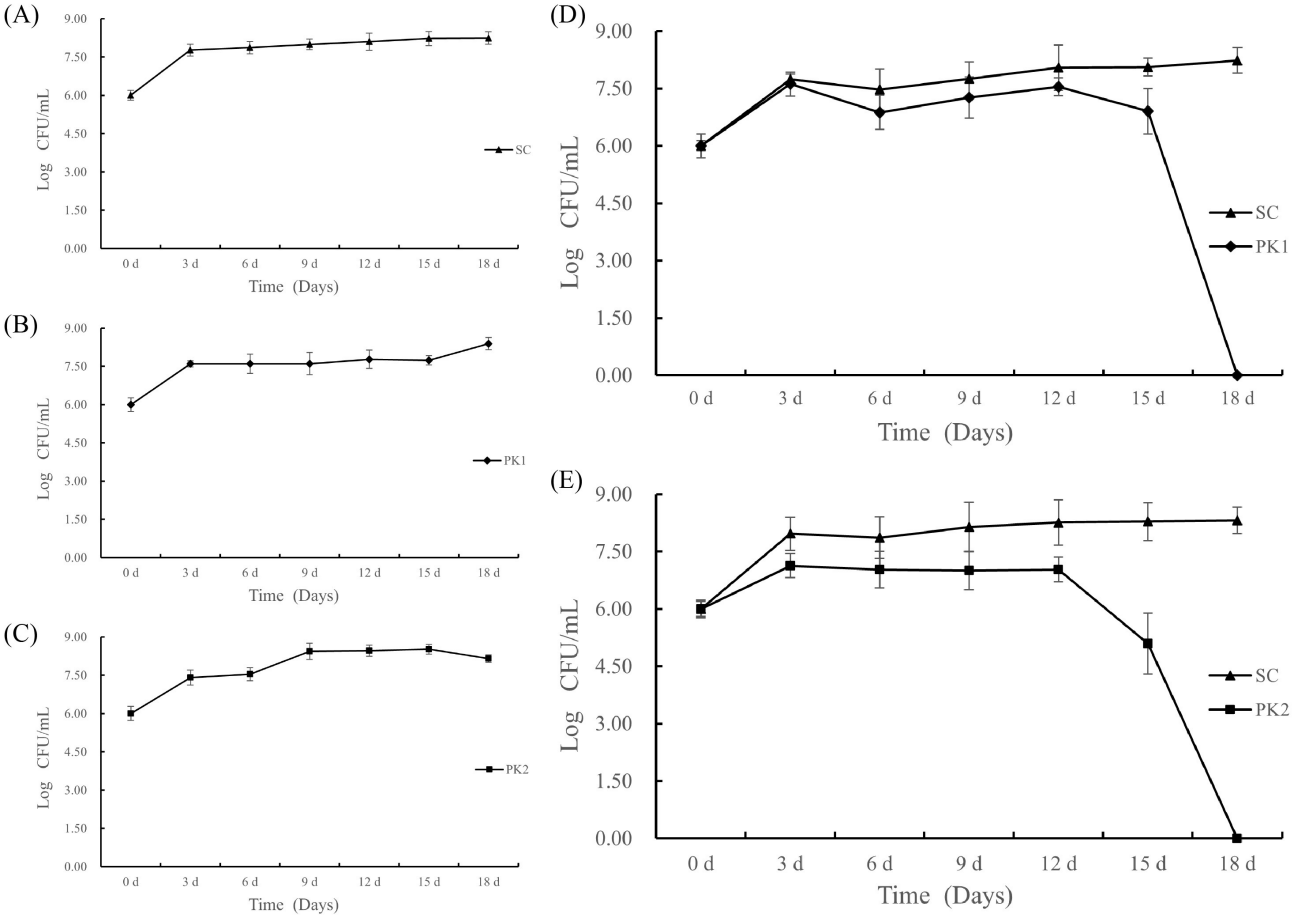
Changes in yeast cell counts (expressed as log CFU/mL) during mulberry wine fermentation. **(A)** Represent pure-fermentation with *S. cerevisiae* (SC), **(B)** Represent pure-fermentation with *P. kluyveri* (PK1), **(C)** Represent pure-fermentation with *P. kluyveri* (PK2), **(D)** Represent co-fermentation with *S. cerevisiae* (SC) and *P. kluyveri* (PK1), **(E)** Represent co-fermentation with *S. cerevisiae* (SC) and *P. kluyveri* (PK2).

The growth kinetics of *S. cerevisia* (SC), *P. kluyveri* (PK1), and *P. kluyveri* (PK2) in pure and co-fermentation were shown in Figure 1. All three pure culture strains showed similar growth trends during the first 3 days. After 3 days, a different trend of slow growth was observed. It was also similar to previous reports from Yu et al. (17). As shown in Figure 1A, the SC cells showed consistent growth after 3 days, eventually reaching the maximum number at 8.25 Log CFU/mL. The PK1 cell population remained stable from day 3 to day 15, followed by a brief increase, reaching a maximum concentration of 8.39 Log CFU/mL at day 18 (Figure 1B). In contrast, increasing numbers of PK2 cells were observed prior to day 9, followed by a relatively short period of stability from day 9 to day 15 (Figure 1C). The number of cells tended to decrease gradually after reaching a maximum of 8.52 Log CFU/mL at 15 d (Figure 1C). For the co-fermentation of SC and PK (PK1 or PK2), the growth of two yeasts were affected by the presence of each other (Figures 1D, E). In the co-fermentation group of F(S-P1) and F(S-P2), the cells of the SC and PK (PK1 or PK2) grew rapidly in the first 3 days and then became essentially stable. After 3 days, the SC strains in group F(S-P1) exhibited a slight improvement in stability, reaching its maximum of 8.23 Log CFU/mL at 18 d. In group F(S-P1), the PK1 strains displayed a declining trend in stability. The PK1 cell population underwent a sharp decline, particularly at day 15, and was no longer detectable by day 18 (Figure 1D).

Correspondingly, the growth kinetics of SC and PK2 strains in group F(S-P2) exhibited patterns similar to those observed in group F(S-P1). However, the PK2 strains exhibited an earlier onset of extinction during co-fermentation, commencing at day 12 and reaching complete depletion by day 18 (Figure 1E). In the F(S-P2) group, the SC strains reached its maximum cell count of 8.31 Log CFU/mL at 18 d, whereas the PK2 strains did not reach its maximum cell count of 7.13 Log CFU/mL until 3 d (Figure 1E). This suggested that the presence of SC influenced the growth of PK (PK1 or PK2) (18). This result might be due to the toxic effect of alcohol and nutritional competition (17). Compared to the PK1, the PK2 was more drastically affected by SC strains.

### 3.2 Classical oenological parameters

The physicochemical characteristics of mulberry juice and wines were shown in Table 1. The final pH values were almost the same in the pure-fermentation broth F(S), F(P1), and F(P2). The co-fermentation groups F(S-P1) and F(S-P2) were also almost identical. Compared to MJ, all fermentation groups improved slightly. The levels of titratable acidity in all fermentation solution samples were between 2.6 and 3.1 g/L. MJ had a relatively low original acidity of only 1.7 g/L in terms of titratable acidity. All samples had a variable increase in acidity after fermentation. An appropriate increase in the acidity level could help to improve the flavor of the fruit wine and make it more mellow (19). In this study the titratable acid of F(S) was found to be only 2.6 g/L, which was close to that of F(P1). It was found that SC and PK1 strains had limited ability to produce acid during fermentation. This resulted in the acid content of co-fermentation F(S-P1) was only 2.7 g/L. However, the PK2 strain was more capable of acid production. The titratable acid of sample F(S-P2) was 3.1 g/L. These results indicated that the acid production capacity of SC and PK (PK1 and PK2) strains in co-fermentation did not have an effect on each other. In comparison with F(S-P1), F(S-P2) had a more acidic taste in the same pH conditions.

**Table 1:** Physicochemical parameters of mulberry juice and mulberry wine (day 18) fermented with *S. cerevisiae* and *P. kluyveri*.

	**MJ**	**F(P1)**	**F(P2)**	**F(S)**	**F(S-P1)**	**F(S-P2)**
pH	3.67 ± 0.00	3.93 ± 0.07	4.01 ± 0.06	3.88 ± 0.06	4.07 ± 0.09	4.00 ± 0.06
SSC (°Brix)	24.67 ± 0.21	18.77 ± 0.12	19.07 ± 0.06	9.00 ± 0.10	8.37 ± 0.12	8.27 ± 0.18
Ethanol content (v/v, %)	ND	2.11 ± 0.09	1.91 ± 0.08	13.26 ± 0.11	11.98 ± 0.10	11.55 ± 0.09
Residual sugars (g/L)	175.39 ± 5.51	90.59 ± 6.44	101.14 ± 7.91	21.08 ± 2.58	24.77 ± 4.21	27.37 ± 5.06
Titratable acidity (g/L)	1.70 ± 0.11	2.70 ± 0.08	3.2 ± 0.13	2.6 ± 0.14	2.70 ± 0.48	3.10 ± 0.41

Values are means ± SE (*n* = 3). Titratable acidity was calculated as malic acid. ND, Not detected.

There was a significant loss of sugar from the mulberry juice in all mulberry wines with SC strains involved in fermentation. The SSC for F(S), F(S-P1), and F(S-P2) were 9, 8.37 and 8.27%, respectively. Correspondingly, the residual sugar content of the three groups of mulberry wines was 21.08, 24.77, and 27.37 g/L, respectively. The large reduction in the sugar content of the three groups of mulberry wines was also reflected in the increased alcohol content. F(S) had the highest alcohol content at 13.26%. The alcohol content of F(S-P1) and F(S-P2) was 11.98 and 11.55%, respectively. This result indicated that the addition of PK strains did not negatively affect the dominance of SC strains in the fermentation. The alcohol-producing capacity of the *S. cerevisiae* still functioned properly in the co-fermentation. It should be noted that co-fermentation produced less alcohol than the pure-fermentation of *S. cerevisiae*. This may be related to the nutrient competition effect caused by non-*Saccharomyces* in the co-fermentation, which prolongs the latency of the *S. cerevisiae* growth process and delays the start of alcoholic fermentation (20).

### 3.3 Analysis of non-volatile compounds

**Figure 2:**
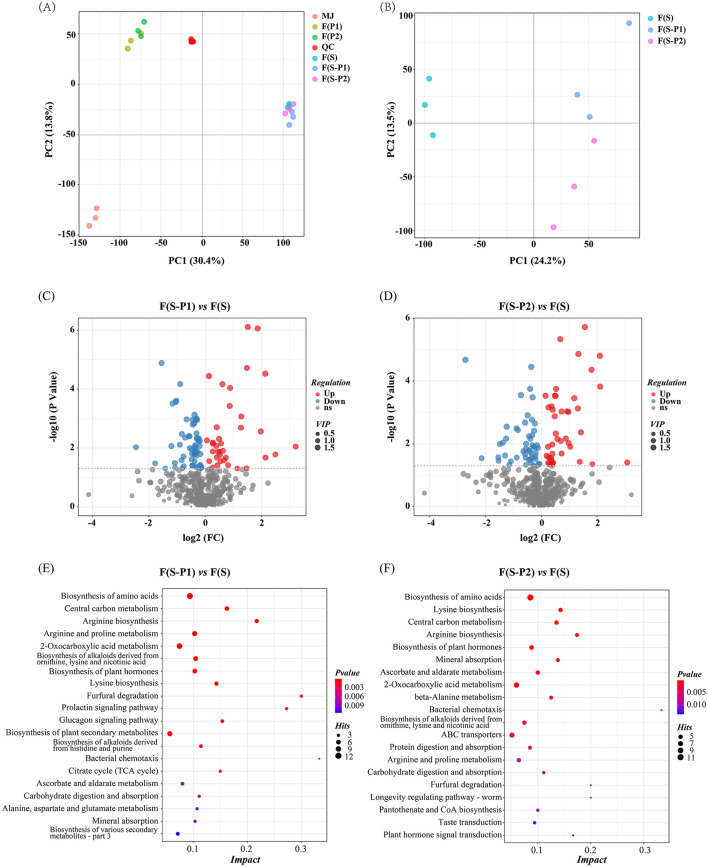
**(A, B)** Principal component analysis of non-volatile components (PCA) score plot of non-volatile components for reliability verification, positive ionization mode; **(C, D)** Volcano plots showing differential metabolites between different mulberry wines (*P* < 0.05, VIP values > 1); **(E, F)** The most prominent pathway (Top 20) enriched KEGG terms among the differential accumulated metabolites (DAMs).

All samples corresponding to different fermentation strains were first visually distinguished using Principal Component Analysis (PCA). As shown in Figure 2A, the quality control (QC) samples had a good tendency to cluster. This was an indication that the analysis was reliable and reproducible. The two principal components expressed about 44.2% of the total variance, with PC1 and PC2 accounting for 30.4 and 13.8%, respectively (Figure 2A).

The results also showed significant differences in the grouping of fermentation broth from different strains. Furthermore, F(P1) and F(P2) metabolite spectra were relatively similar and clustered together. Instead, F(S-P1) and F(S-P2) had been clustered together with F(S). Two specimen sets showed good separation at PC1. This result indicated that the fermenting broth of SC strains dominated the grouping. With a further focus on three types of mulberry wine F(S), F(S-P1), and F(S-P2), as shown in Figure 2B, all wine samples showed clear separation. The first component (PC1, 24.2%) separated F(S) from other co-fermentation samples. The F(S-P1) and F(S-P2) samples were clearly separated by the second component (PC2, 13.5 %). F(S) was clearly separated from the other samples and held a dominant position, as shown in Figure 2B.

The differing patterns of metabolite accumulation could be clearly visualized using clustering heatmaps (Supplementary Figure S1). The results showed significant differences in metabolites between different samples and clear clusters were formed. The clustering heatmap also showed good clustering between different biological replicates. Meanwhile, the clustering trend of the samples was consistent with the PCA (Figure 2B) conclusion. The three types of mulberry wines [F(S), F(S-P1), and F(S-P2)] appeared to be clustered, with F(S) occupying a dominant position (Figure 2A). All metabolites detected in Supplementary Figure S1 were listed in Supplementary Table S1.

Finally, the focus was on the differences between pure fermentation (SC) and co-fermentation (with PK1 or PK2) of mulberry wine. The *P* < 0.05 and VIP values >1 were considered to be statistically significant metabolites (Figures 2C, D). By comparing F(S-P1) and F(S), a total of 97 differential metabolites were identified (Figure 2C, Supplementary Table S2, and Supplementary Figure S2). On the other hand, In the comparison of F(S-P2) and F(S), 94 different metabolites were identified (Figure 2D, Supplementary Table S3, and Supplementary Figure S3).

To further identify metabolic pathways in the different mulberry wines, the differential accumulated metabolites (DAMs) were mapped in the KEGG database. KEGG functional enrichment analysis was also performed to assess the presence and distribution of DAMs (Figures 2E, F). Amino acid biosynthesis or metabolism were the most prominent pathway enriched KEGG term among the DAMs detected for all compared samples. Given the unique role of amino acids in the flavor of fruit wine (17), it was reasonable to speculate that DAMs in the amino acids biosynthesis or metabolism pathway might be the key reason leading to flavor changes in the co-fermentation of mulberry wine. These results underlined the importance of these compounds in the flavor differences of co-fermented mulberry wine.

**Figure 3:**
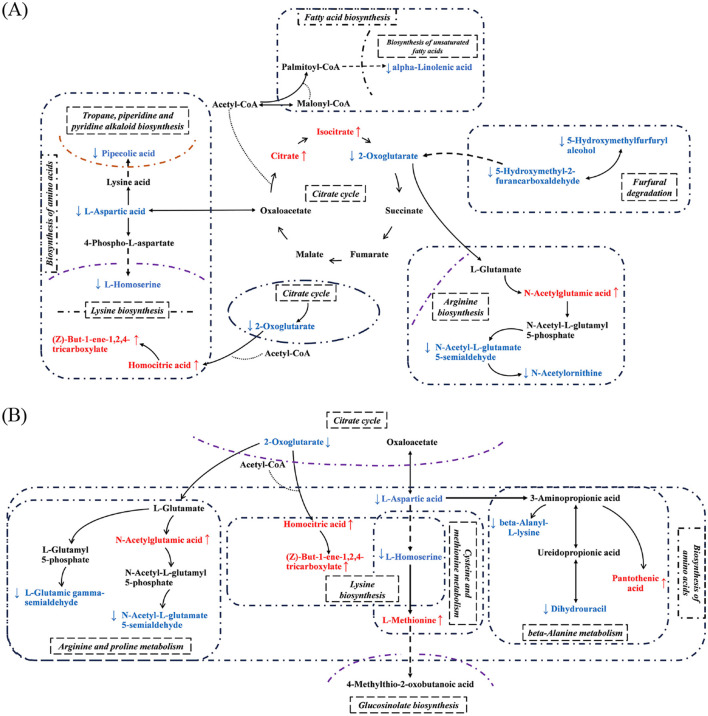
Integration map of prominent pathway. **(A)** F(S-P1), **(B)** F(S-P2). The arrow pointing upwards indicates increased in concentration levels; The arrow pointing downwards indicates decreased in concentration levels.

The key pathways involved in the significant differential metabolites (SDMs) provided a visual demonstration of the differences between the different co-fermentation strategies (Figure 3). The initial metabolism of the SC strains was under the influence of the co-fermentation. The PK strains significantly disturbed the TCA cycle and the corresponding amino acid metabolism as shown in Figure 3. In comparison to the PK2 strains, the PK1 strains had a greater effect on the TCA cycle, which in turn had a more extensive global effect on amino acid metabolism, fatty acid metabolism and other processes (Figure 3A). The PK2 strains, however, mainly improved the metabolism of amino acids. Several pathways of amino acid metabolism were involved in SDMs (Figure 3B). Furthermore, the comparison showed that SDMs in the different co-fermentation strategies could be traced to substances, L-aspartic acid and 2-oxoglutarate. Coincidentally, both substances showed a downward trend in different co-fermentation strategies. The amino acid pathways involved in 2-Oxoglutarate became active and the corresponding TCA cycle was disturbed. This also explained the accumulation of citrate and isocitrate in the TCA pathway of F(S-P1). But this phenomenon did not appear at F(S-P2). In addition, L-aspartic acid is a key substance for the formation of other amino acids by micro-organisms (21). This also confirmed the decreased concentration of L-asparagine in co-fermentation, possibly due to the overall high level of amino acid metabolism. The amino acids are precursors for many flavor compounds (22). It is possible that the PK strain improved amino acid metabolism during the co-fermentation process, which contributed to a significant increase in the levels of aroma compounds and an improvement in the flavor type of mulberry wine.

### 3.4 Volatile flavor compounds of mulberry wine

**Figure 4:**
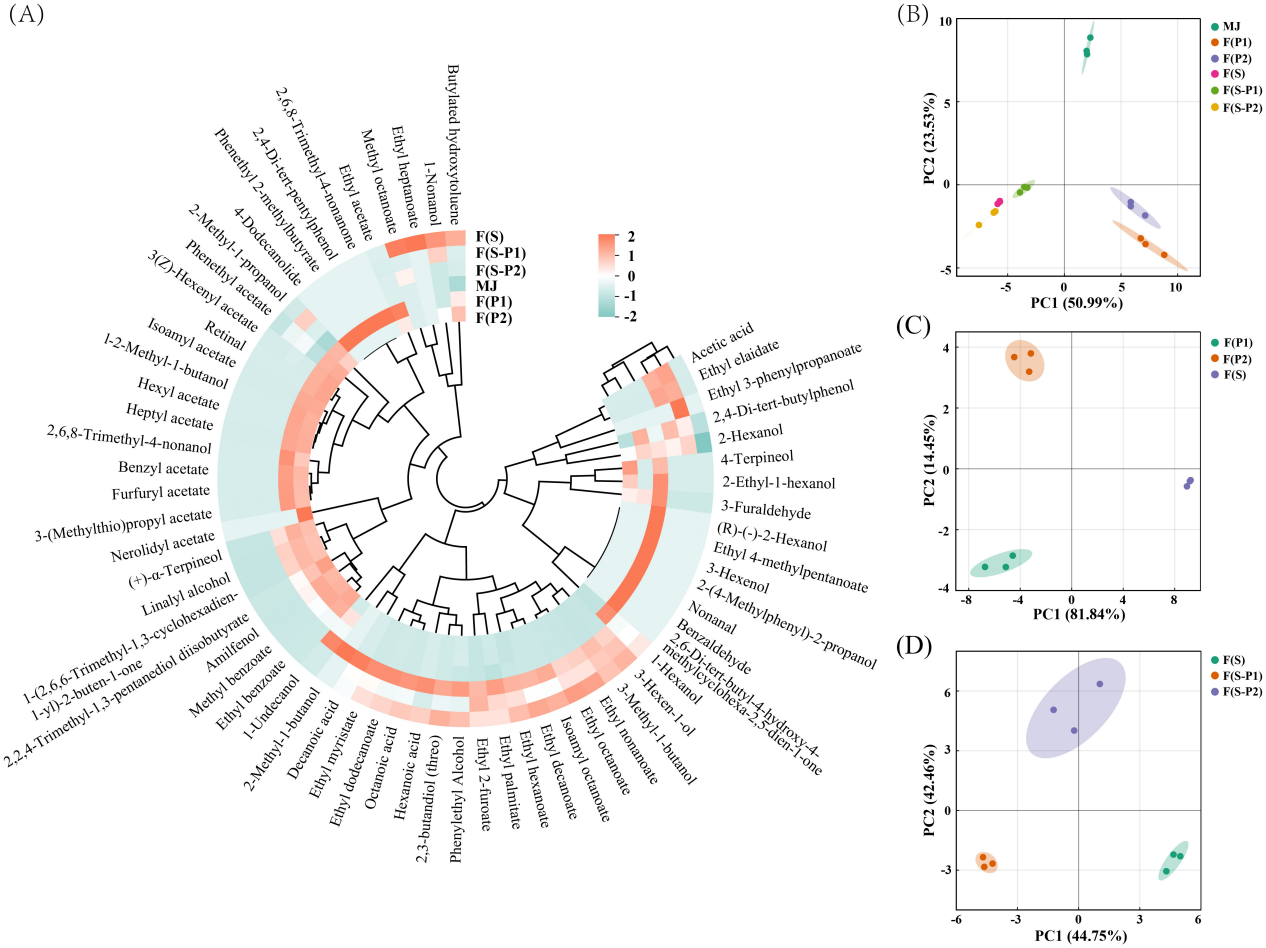
**(A)** Heatmap of characteristic volatile compounds in different mulberry fermentation broth (18 d), **(B–D)** Principal component analysis (PCA) score plot of volatile components.

After fermentation with pure or co-culture, as shown in Figure 4A, the types and levels of volatile flavor compounds varied considerably. The contents of identified volatiles were presented in Table 2. A total of 63 volatiles were detected in different fermentation broths, as shown in Figure 4. The volatile compounds included 29 esters, 19 alcohols, and 3 ketones compounds. There were four each of acids, aldehydes and others.

**Table 2:** Volatile compounds (μg/mL) from mulberry juice and mulberry wine (day 18) fermented with *S. cerevisiae* and *P. kluyveri*.

**No**.	**Compounds**	**CAS**	**Odor description**	**RI**	**Samples**					
					**MJ**	**F(P1)**	**F(P2)**	**F(S)**	**F(S-P1)**	**F(S-P2)**
**Esters**										
1	Methyl benzoate	93-58-3	Prune, lettuce, herb, sweet	1,547	0.47 ± 0.02^a^	1.32 ± 0.28^b^	1.24 ± 0.21^b^	ND	ND	ND
2	Ethyl benzoate	93-89-0	Camomile, flower, celery, fruit	1,595	0.54 ± 0.16^a^	1.80 ± 0.71^b^	1.90 ± 0.39^b^	ND	ND	ND
3	Phenethyl acetate	103-45-7	Rose, floral	1,736	2.42 ± 0.50^a^	402.96 ± 41.15^c^	421.51 ± 78.04^c^	25.86 ± 1.79^a^	141.71 ± 8.48^b^	166.86 ± 17.82^b^
4	Ethyl heptanoate	106-30-9	Pineapple, fruity	1,285	ND	ND	ND	0.27 ± 0.01	ND	ND
5	Ethyl octanoate	106-32-1	Floral, fruity, banana, pear, brandy, sweet	1,388	ND	0.76 ± 0.09^a^	0.85 ± 0.15^a^	121.48 ± 8.39^c^	66.25 ± 3.93^b^	77.81 ± 16.99^b^
6	Ethyl dodecanoate	106-33-2	Floral, fruit, green apple, leaf, nut	1,798	ND	0.34 ± 0.05^a^	0.27 ± 0.05^a^	13.55 ± 0.14^c^	5.22 ± 1.05^b^	23.88 ± 5.73^d^
7	Ethyl decanoate	110-38-3	Fruity, grape, brandy	1,598	ND	ND	ND	133.15 ± 6.57^c^	49.51 ± 5.77^a^	120.33 ± 13.49^b^
8	Methyl octanoate	111-11-5	Orange	1,339	ND	ND	ND	0.42 ± 0.03^b^	ND	0.13 ± 0.02^a^
9	Heptyl acetate	112-06-1	floral, fresh	1,330	ND	0.34 ± 0.14^a^	0.32 ± 0.05^a^	ND	ND	ND
10	Ethyl nonanoate	123-29-5	banana, fruit, grape	1,488	ND	ND	ND	1.71 ± 0.21^b^	0.97 ± 0.19^a^	1.21 ± 0.23^a^
11	Ethyl hexanoate	123-66-0	Fruity, green apple, banana, brandy, wine-like	1,193	0.28 ± 0.19^a^	ND	ND	10.10 ± 1.89^c^	7.34 ± 0.99^b^	10.88 ± 2.26^c^
12	Isoamyl acetate	123-92-2	Banana, fruity, sweet	1,086	ND	138.92 ± 8.15^a^	137.05 ± 7.36^a^	ND	ND	ND
13	Ethyl myristate	124-06-1	Lily	2,003	ND	ND	ND	3.90 ± 0.40^a^	2.71 ± 0.84^a^	7.81 ± 1.95^b^
14	Benzyl acetate	140-11-4	Boiled vegetable	1,652	ND	0.75 ± 0.09^b^	0.62 ± 0.12^a^	ND	ND	ND
15	Ethyl acetate	141-78-6	Pineapple	745	2.19 ± 0.10^a^	49.29 ± 3.37^c^	19.02 ± 3.60^b^	2.80 ± 0.70^a^	1.48 ± 0.23^a^	2.63 ± 0.39^a^
16	Hexyl acetate	142-92-7	Fruity, herb	1,233	ND	4.13 ± 0.77^a^	3.84 ± 0.80^a^	ND	ND	ND
17	Ethyl 2-furoate	614-99-3	-	1,545	ND	ND	ND	2.07 ± 0.53^a^	3.10 ± 0.63^b^	3.00 ± 0.54^b^
18	Furfuryl acetate	623-17-6	-	1,463	ND	0.56 ± 0.15^b^	0.44 ± 0.05^a^	ND	ND	ND
19	Ethyl palmitate	628-97-7	Waxy, greasy	2,211	0.44 ± 0.13^a^	4.94 ± 0.68^b^	5.64 ± 0.48^b^	25.81 ± 3.57^c^	27.97 ± 1.62^c^	43.09 ± 4.22^d^
20	Ethyl 3-phenylpropanoate	2021-28-5	Floral	1,702	ND	ND	ND	ND	1.68 ± 0.37	ND
21	Isoamyl octanoate	2035-99-6	-	1,620	ND	ND	ND	3.05 ± 0.24^b^	1.18 ± 0.13^a^	3.18 ± 0.40^b^
22	4-Dodecanolide	2305-05-7	Fruity, flower, sweet	1,942	ND	0.37 ± 0.05	ND	ND	ND	ND
23	Nerolidyl acetate	2306-78-7	-	1,975	ND	ND	0.20 ± 0.04	ND	ND	ND
24	3(Z)-Hexenyl acetate	3681-71-8	Banana	1,270	ND	8.69 ± 2.28^b^	9.45 ± 1.50^b^	ND	ND	0.34 ± 0.02^a^
25	Ethyl elaidate	6114-18-7	-	2,428	ND	ND	ND	ND	1.77 ± 0.13^a^	1.86 ± 0.22^a^
26	2,2,4-Trimethyl-1,3-pentanediol diisobutyrate	6846-50-0	-	1,820	0.41 ± 0.01 a	0.79 ± 0.06^b^	1.02 ± 0.14^c^	ND	ND	ND
27	3-(Methylthio)propyl acetate	16630-55-0	-	1,557	ND	0.71 ± 0.13^b^	0.55 ± 0.09^a^	ND	ND	ND
28	Phenethyl 2-methylbutyrate	24817-51-4	-	1,802	ND	3.43 ± 0.84	ND	ND	ND	ND
29	Ethyl 4-methylpentanoate	25415-67-2	Fruity	1,197	0.07 ± 0.01	ND	ND	ND	ND	ND
**Alcohols**										
1	Phenylethyl Alcohol	60-12-8	Rose, honey	1,815	0.89 ± 0.16^a^	6.27 ± 1.27^ab^	5.28 ± 1.22^ab^	43.09 ± 4.36^c^	12.14 ± 2.19^b^	55.51 ± 8.73^d^
2	2-Methyl-1-propanol	78-83-1	Wine, solvent, bitter	1,096	ND	3.58 ± 1.08^c^	3.04 ± 0.46^c^	0.90 ± 0.21^ab^	3.04 ± 0.49^c^	1.13 ± 0.08^b^
3	Linalyl alcohol	78-70-6	Flower, lavender	1,489	0.70 ± 0.06^a^	0.84 ± 0.22^a^	0.78 ± 0.12^a^	ND	ND	ND
4	2-Ethyl-1-hexanol	104-76-7	Rose	1,436	0.77 ± 0.12^b^	ND	0.47 ± 0.08^a^	ND	ND	ND
5	1-Hexanol	111-27-3	Floral	1,298	1.93 ± 0.08^c^	ND	ND	0.96 ± 019^b^	0.71 ± 0.09^a^	0.60 ± 0.11^a^
6	1-Undecanol	112-42-5	Mandarin, green	1,604	0.60 ± 0.17^a^	1.28 ± 0.15^b^	ND	ND	ND	2.91 ± 0.64^c^
7	2,6,8-Trimethyl-4-nonanol	123-17-1	-	1,523	ND	0.78 ± 0.21^b^	0.50 ± 0.09^a^	ND	ND	ND
8	3-Methyl-1-butanol	123-51-3	Burnt, alcohol, nail polish, whiskey	1,178	ND	ND	ND	143.66 ± 12.46^a^	135.41 ± 6.84^a^	147.64 ± 15.90^a^
9	2-Methyl-1-butanol	137-32-6	Wine, onion	1,177	ND	ND	ND	ND	ND	16.75 ± 2.89
10	1-Nonanol	143-08-8	Fat, green	1,605	ND	ND	1.60 ± 0.23^a^	4.80 ± 0.64^c^	3.03 ± 0.76^b^	ND
11	3-Hexenol	544-12-7	-	1,203	2.37 ± 0.06	ND	ND	ND	ND	ND
12	4-Terpineol	562-74-3	Turpentine, nutmeg, must	1,541	0.27 ± 0.01^a^	ND	0.34 ± 0.05^b^	ND	ND	ND
13	2-Hexanol	626-93-7	-	1,138	30.18 ± 1.00^a^	32.67 ± 10.58^a^	24.10 ± 4.55^a^	ND	33.00 ± 0.87^a^	27.15 ± 5.36^a^
14	3-Hexen-1-ol	928-96-1	Grass	1,324	ND	ND	ND	0.71 ± 0.04^a^	0.63 ± 0.14^a^	0.55 ± 0.16^a^
15	2-(4-Methylphenyl)-2-propanol	1197-01-9	Citrus, must	1,769	0.21 ± 0.06	ND	ND	ND	ND	ND
16	l-2-Methyl-1-butanol	1565-80-6	Malt	1,170	ND	10.29 ± 0.49^b^	9.33 ± 0.34^a^	ND	ND	ND
17	(+)-α-Terpineol	7785-53-7	-	1,635	0.25 ± 0.03^a^	0.39 ± 0.07^b^	0.34 ± 0.07^b^	ND	ND	ND
18	2,3-butandiol (threo)	19132-06-0	-	1,465	ND	ND	ND	2.08 ± 0.19^b^	0.48 ± 0.18^a^	2.21 ± 0.16^b^
19	(R)-(-)-2-Hexanol	26549-24-6	-	1,149	4.79 ± 0.06	ND	ND	ND	ND	ND
**Acids**										
1	Benzaldehyde	100-52-7	Almond	1,448	0.39 ± 0.06	ND	ND	ND	ND	ND
2	Retinal	116-31-4	-	1,949	ND	0.08 ± 0.01^a^	0.09 ± 0.01^a^	ND	ND	ND
3	Nonanal	124-19-6	Citrusy, floral	1,349	0.85 ± 0.21	ND	ND	ND	ND	ND
4	3-Furaldehyde	498-60-2	-	1,388	2.79 ± 0.38^b^	1.35 ± 0.46^a^	1.15 ± 0.29^a^	ND	ND	ND
**Aldehydes**										
1	2,6,8-Trimethyl-4-nonanone	123-18-2	-	1,363	ND	0.35 ± 0.09	ND	ND	ND	ND
2	2,6-Di-tert-butyl-4-hydroxy-4-methylcyclohexa-2,5-dien-1-one	10396-80-2	-	2,025	0.29 ± 0.12	ND	ND	ND	ND	ND
3	1-(2,6,6-Trimethyl-1,3-cyclohexadien-1-yl)-2-buten-1-one	23696-85-7	-	1,751	0.44 ± 0.05^a^	0.56 ± 0.07^b^	0.51 ± 0.10^ab^	ND	ND	ND
4	3-Furaldehyde	498-60-2	-	1,388	2.79 ± 0.38^b^	1.35 ± 0.46^a^	1.15 ± 0.29^a^	ND	ND	ND
**Ketones**										
1	2,6,8-Trimethyl-4-nonanone	123-18-2	-	1,363	ND	0.35 ± 0.09	ND	ND	ND	ND
2	2,6-Di-tert-butyl-4-hydroxy-4-methylcyclohexa-2,5-dien-1-one	10396-80-2	-	2,025	0.29 ± 0.12	ND	ND	ND	ND	ND
3	1-(2,6,6-Trimethyl-1,3-cyclohexadien-1-yl)-2-buten-1-one	23696-85-7	-	1,751	0.44 ± 0.05^a^	0.56 ± 0.07^b^	0.51 ± 0.10^ab^	ND	ND	ND
**Others**										
1	Amilfenol	80-46-6	NM	2,307	0.08 ± 0.01^a^	0.15 ± 0.02^b^	0.14 ± 0.02^b^	ND	ND	ND
2	2,4-Di-tert-butylphenol	96-76-4	NM	2,228	23.82 ± 3.43^a^	66.78 ± 3.73^c^	ND	ND	39.85 ± 6.34^b^	61.89 ± 7.00^c^
3	2,4-Di-tert-pentylphenol	120-95-6	NM	2,384	ND	0.08 ± 0.00	ND	ND	ND	ND
4	Butylated Hydroxytoluene	128-37-0	NM	1,849	1.90 ± 0.59^a^	11.89 ± 0.46^c^	15.94 ± 0.95^d^	17.53 ± 1.34^d^	6.62 ± 0.94^b^	6.27 ± 1.08^b^

Odor thresholds were taken from website vcf-online and flavornet (www.vcf-online.nl, www.flavornet.org).

Values are means ± SE (*n* = 3). ND, Not detected.

Statistically significant differences between different sample are shown with lower case letters a–d (ANOVA with Tukey's test, *p* < 0.05).

In order to further identify the differences in the volatiles of the different samples, a principal components analysis (PCA) was carried out. As shown in Figure 4B, all samples were clearly separated from each other. In addition, the PCA results also exactly indicated that the fermentation with pure and co-culture of yeast would be distinguished (Figure 4D). In Figure 4D, the F(S) group was divided by the positive PC1 scores, and the F(S-P1) group was divided by the negative PC1 scores. On the other hand, the F(S-P2) could be separated by the scores of PC2. The classification trend of the different fermentation broths was also shown by the PCA results (Figures 4C, D). The PC1 results (81.84%, 44.75%) in Figures 4C, D initially showed the dominance of SC strains in fermentation. In Figure 4C, PK1 and PK2 could be separated by second component (PC2, 14.45 %). When the PK strains were used in a co-fermentation, the F(S-P1) and F(S-P2) could also be well separated by the second component (PC2, 42.46%) and showed significant differences (Figure 4D). This was an indication that the differences between PK1 strain and PK2 strain could be amplified during co-fermentation with SC strain. In other words, the fermentation process was dominated by the SC strain, and PK strains formed the flavor of the different types of mulberry wine.

The samples of mulberry juice (MJ) had a simple volatile composition and low levels of the individual components. This was similar to the insignificant flavor of the mulberry fruit. After fermentation by different strains, seven substances in MJ were not found. These were mainly aldehydes (nonanal and benzaldehyde) and some heterocyclic compounds. This result was in agreement with a previous study (23).

The esters are one of the main aroma components in fermentation, creating the main flavor type of fruit wine (24). The total amount of esters produced in F(P1) and F(P2) was high, and their relative content among all the components was also relatively high (Figure 4A, Table 2). This indicated the excellent ester production performance of the PK1 and PK2 strains, similar to previous reports (25). Phenethyl acetate was the most abundant of all the esters detected, with 402.96 μg/mL and 421.51 μg/mL detected in F(P1) and F(P2), respectively. Similarly, phenethyl acetate was also the ester with the highest content in F(S-P1) and F(S-P2), reaching 141.71 and 166.86 μg/mL, respectively. The content of phenethyl acetate in the F(S) was only 25.86 μg/mL. This suggested that phenethyl acetate was responsible for a “rose” and “floral” odor (24) in co-fermented mulberry wines. In other words, these results indicated that co-fermentation completely changed the flavor type of fruit wine. On the other hand, the content of ethyl decanoate and ethyl octanoate in F(S) reached 133.15 and 121.48 μg/mL, respectively, which was the highest of all groups. This also constructed the “brandy” odor as the main flavor type in F(S). Comparing F(S-P1) and F(S-P2), it was found that there was a significant difference in the content of ethyl palmitate, ethyl myristate, ethyl dodecanoate, isoamyl octanoate, and ethyl decanoate (Figure 4A). This also explained the flavor differences between the co-fermentation groups.

Although alcohols were found in all groups, the types and amounts of alcohols in MJ, F(P1) and F(P2) were on low levels. The alcohol content increased significantly and its type changed after fermentation (SC involved), becoming one of the most important components in the resulting mulberry wines (Figure 4A). It should be noted that 3-Methyl-1-butanol was significantly increased in the F(S) and co-fermentation groups, whereas it was not found in the MJ and non-*Saccharomyces* fermentation groups F(P1) and F(P2). This indicated that the production of 3-Methyl-1-butanol came mainly from the metabolism of the SC strain. The addition of the PK1 and PK2 strains had no significant effect on it. The presence of 3-Methyl-1-butanol also enhanced the “floral” and “nail polish” flavors (26) of mulberry wine. High concentration of phenylethyl alcohol was found in F(S-P2) from the co-fermentation, followed by that from F(S). This also contributed to the “floral” and “honey” flavors of mulberry wines.

The acids were mostly produced during fermentation (27). The content of octanoic acid (65.98 μg/mL) and decanoic acid (32.16 μg/mL) in F(S-P2) is significantly higher than in F(S) and F(S-P1). However, no acid of any kind was found in the F(P1) and F(P2). This means that the SC strain could produce these two acids, while the PK2 strain helped to increase their production during co-fermentation process. In F(S-P1), on the other hand, octanoic acid had only 16.41 μg/mL and hexanoic acid could not be detected. This suggests that PK1 strain inhibited both acids during co-fermentation. At least the influence of metabolic mechanisms between SC and PK (PK1 and PK2) strains was clearly demonstrated by the conclusion of titratable acidity (TA). In particular, acetic acid was not found in F(S). This indicated that the SC strain was effective in inhibiting acetic acid production.

The aldehydes were not found in either pure cultured F(S) or co-fermented [F(S-P1) and F(S-P2)] mulberry wines containing SC strain. The amount and content of aldehydes in F(P1) and F(P2) were lower than in MJ. This result was in line with the findings of the study by YU et al. (17). Similar results were obtained for ketones. In particular, no ketones were detected in F(S) or in the co-fermentations of F(S-P1) and F(S-P2). Previous studies have shown that it had little effect on the flavor of fruit wine (14).

### 3.5 Comprehensive metabolic characteristic analyses

Amino acid metabolism in the co-fermentation system showed very active with the addition of the PK strain. The formation of various amino acids was also based on this. The positive effect of amino acid metabolism on fermentation was confirmed (28). The amino acids were essential nutrients for the growth and metabolism of yeast (29). In addition, some of the amino acids could be metabolized by the yeast (both SC and PK strains) to form higher alcohols (30). The PK strain in this study showed excellent growth characteristics during co-fermentation compared to previous studies (18, 25). The PK1 and PK2 strains gradually disappeared after 15 days. This was relatively rare in co-fermentation with SC strains. In general, the growth of yeast has a certain preference in terms of amino acid requirements (29). To maintain strain cell growth, *P. kluyveri* strains rely on amino acids such as Asp and Phe (31). In this study, we observed a significant decreased concentration of the Asp and Phe (Tab S2, S3). This was an indication that the PK1 and PK2 strains may have effectively utilized these two types of nutrients for growth. From this we concluded that the co-fermentation of *P. kluyveri* and *S. cerevisiae* objectively improves biosynthesis of amino acids (Figure 3), which improved the growth status of the *P. kluyveri*. This could be related to the characteristics of the strains and the fermentation temperature (16°C) we chose, but further genetic evidence was needed.

On the other hand, there was also a high demand for amino acid nutrients for the growth of SC strains. In this study, the SC strain was able to maintain an excellent growing state and dominate the co-fermentation process. Obviously, it also benefits from adequate amino acid nutrition (Figures 2E, F, Supplementary Tables S2, S3). In particular, the SC strains were able to maintain higher vitality in the later stages (12–18 d) of co-fermentation. In addition to the positive effects brought about by an active pathway of biosynthesis of amino acids (Figures 2E, F), the dead PK strains were also able to passively provide more amino acids to the SC by autolysis due to the accumulation of large numbers of PK cells.

Generally, most higher alcohols are derived from the anabolic pathway of sugars (32). The metabolomics results in this study showed that the co-fermentation group had a significantly up-regulated central carbon metabolism compared to the F(S) group (Figures 2E, F). Amino acid metabolism was also found to play an important role in flavoring accumulation (21). As an example, some amino acids can be catabolized through the Ehrlich pathway to the given higher alcohols and subsequently to higher alcohol acetates (32). In line with this, we found that substances such as 2-Hexanol and 2-Methyl-1-butanol were only present in the co-fermentation group. Therefore, there was every reason to believe that PK strains involved in co-fermentation would lead to active central carbon metabolism, inevitably accumulating more acetic acid, acetyl-CoA and higher alcohols, which could form higher alcohol esters.

We found that there was a certain difference in the accumulation of higher alcohols between the co-fermenting group and the pure culture group, but it was not very significant (Figure 4). The corresponding ester substances, however, increased significantly (Figure 4). For example, the content of phenylethyl alcohol in F(S) and F(S-P1) was 43.09 and 12.14 μg/mL, respectively. While phenethyl acetate levels reached 25.86 and 141.71μg/mL, respectively (Table 2). In particular, similar results were also observed in the F(S-P2). There are two main pathways by which yeast can produce phenethyl acetate, according to a previous study (33). The first is the synthesis from acetic acid and phenylethyl alcohol under the promotion of esterase. Secondly, it is synthesized from acetyl-CoA and phenylethyl alcohol under the promotion of alcohol acyltransferase (AAT). We further illustrated that PK strain could strongly promote given amino acid (L-phenylalanine, etc.) induced ethanol dehydrogenase (ADH) for the conversion of ethanol to acetic acid in co-fermentation. At the same time, promoted the formation of phenethyl acetate from phenylethyl alcohol and acetyl-CoA via the AAT pathway. In other words, PK strains could promote the formation of more higher alcohol acetates through the AAT pathway in the co-fermentation process. This is all due to the PK strain promoting amino acid metabolism in the co-fermentation system. Correspondingly, we also found that a certain amount of acetic acid was accumulated in the F(S-P1) and F(S-P2), whereas it was not detected in the F(S) group (Table 2). The accumulation of acetic acid also supports our view to some extent. However, this hypothesis is in need of confirmation by further research on enzyme activity.

## 4 Conclusion

In summary, this study provides insights into the co-fermentation of *S. cerevisiae* with different *P. kluyveri* strains and offers a novel fermentation strategy for expanding the flavor profiles of fruit wines. The PK strains, by modulating amino acid metabolism during co-fermentation, impart unique “floral” and “fruity” aromas to mulberry wine, particularly the “rose-like” flavor contributed by ester compounds. The different PK strains improved the levels of aroma categories and types in the co-fermented mulberry wine to varying degrees. Investigating the effect of PK strains on the metabolic progression during co-fermentation will further enable the relatively precise prediction and control of aromatic profiles in fruit wines for practical production applications.

## Data Availability

The original contributions presented in the study are included in the article/Supplementary material, further inquiries can be directed to the corresponding authors.
