# Supplementary material for: Impact of co-fermentation of Saccharomyces cerevisiae and Pichia kluyveri on the metabolic characteristics of the flavor compounds in mulberry wine
Source: Front Nutr. 2025 Feb 25;12:1559599. doi: 10.3389/fnut.2025.1559599 (PMC11893436; doi:10.3389/fnut.2025.1559599)
Supplement: Supplementary file 2 [file Image_1.pdf]

Supplementary Material

1 Supplementary Figures

1.1 Supplementary Figures 1

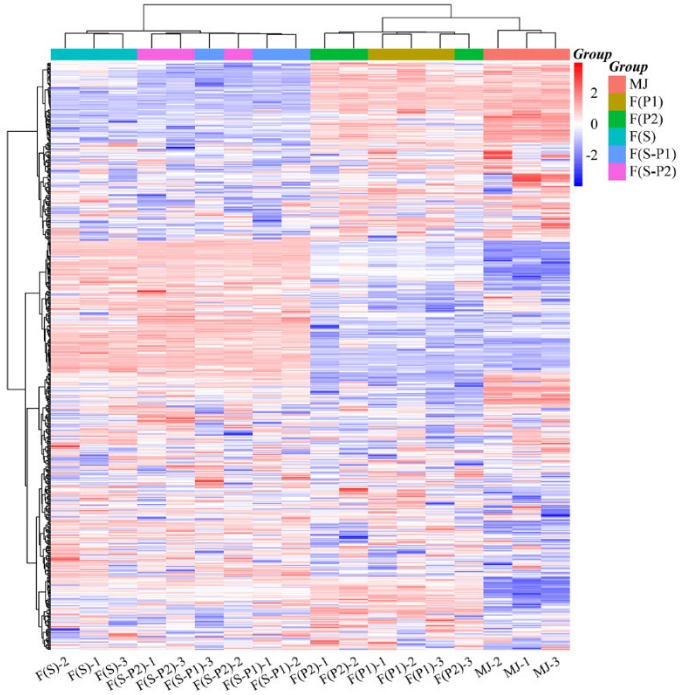

Supplementary Figure 1. Heatmap of characteristic non-volatile compounds in different mulberry wines (18 d).

1.2 Supplementary Figures 2

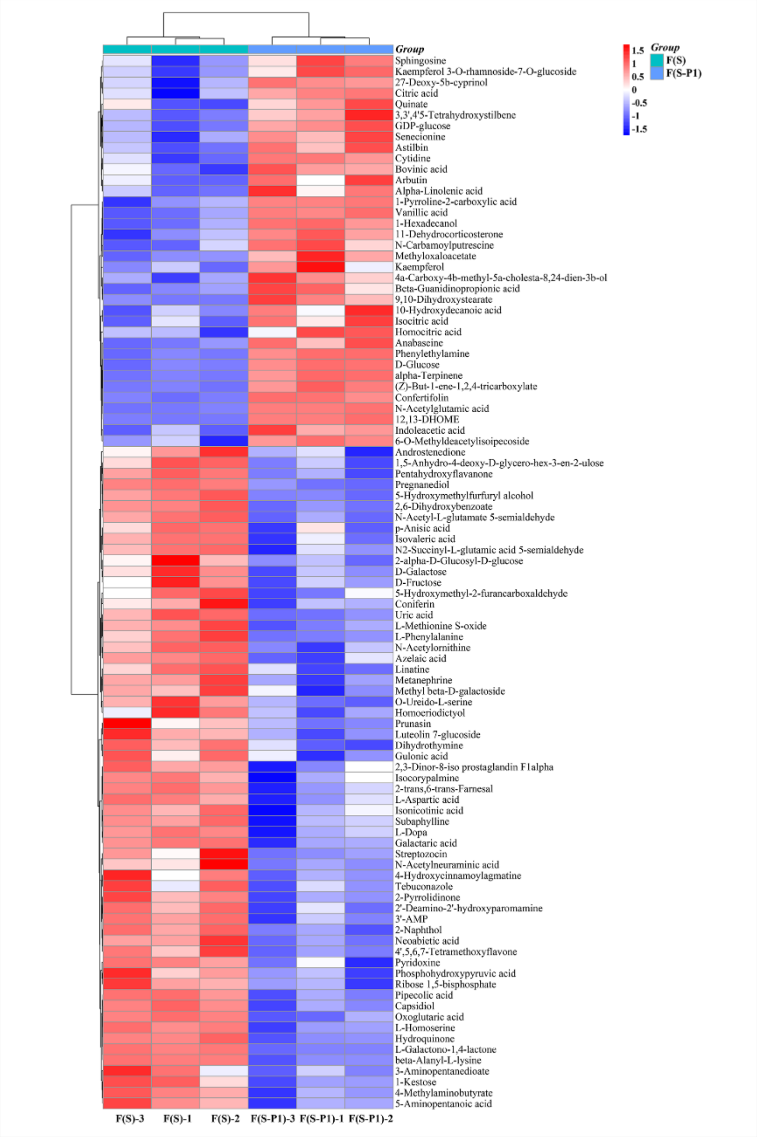

Supplementary Figure 2. Differential metabolites between F(S-P1) and F(S).

1.3 Supplementary Figures 3

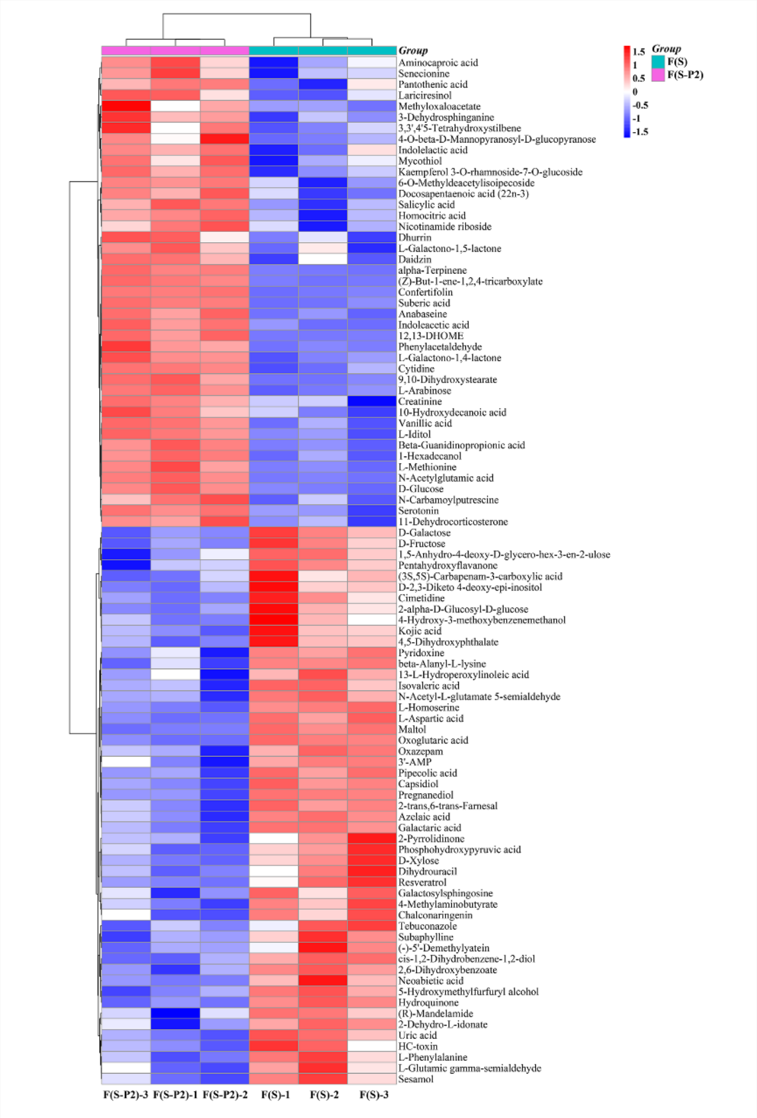

Supplementary Figure 3. Differential metabolites between F(S-P2) and F(S).
